# Surface modification of a BN/ETDS composite with aniline trimer for high thermal conductivity and excellent mechanical properties

**DOI:** 10.1039/c8ra03875a

**Published:** 2018-06-21

**Authors:** Seokgyu Ryu, Taeseob Oh, Jooheon Kim

**Affiliations:** School of Chemical Engineering & Materials Science, Chung-Ang University Seoul 06974 Republic of Korea jooheonkim@cau.ac.kr

## Abstract

Boron nitride (BN) particles surface-treated with different amounts of aniline trimer (AT) were used to prepare thermally conductive polymer composites with epoxy-terminated dimethylsiloxane (ETDS). For the same weight content of BN, the BN composites surface-treated with AT showed better mechanical strength and thermal conductivity than the pure BN composites. This is because of the intercalation of AT between BN and ETDS, which not only increased the wettability but also provided excellent heat transfer pathways. We determined the optimum surface treatment ratio by varying the amount of AT, and the results are discussed regarding the thermal conductivity, storage modulus, and tensile strength. Finally, we established the optimum AT ratio for BN surface treatment.

## Abstract 2

Boron nitride (BN) particles surface-treated with different amounts of aniline trimer (AT) were used to prepare thermally conductive polymer composites with epoxy-terminated dimethylsiloxane (ETDS).

## Graphical abstract

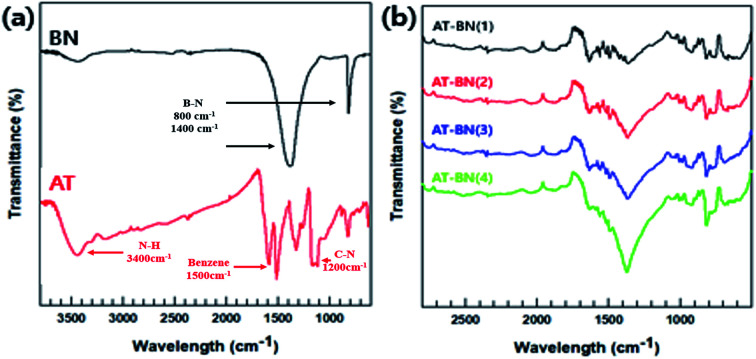

## 1. Introduction

As the demand for high density and fast circuits increases, the need for heat dissipation in electronic packaging has increased correspondingly. A significant amount of heat is generated during the operation of microelectronic products and must be released to prevent overheating. A slight variation in the junction temperature of the electric device can halve its lifetime. Therefore, to ensure the durability and stability of next-generation electronic devices, proper heat dissipation is essential during operation.^1–3^ A low thermal resistance between the electronic device surface and the heat sink is required; however, a substantial portion of the thermal resistance of the device depends on the interfacial morphology (*i.e.*, interstitial gaps). A thermal interface material (TIM) can be used to reduce the rough structure and gaps at the interface. TIMs increase the thermal conductivity by reducing the interfacial thermal resistance on the solid surface between the microprocessor and the heat sink, thus increasing the efficiency of heat transfer. In addition, TIMs conduct heat more efficiently than air, reducing the resistance to heat transfer in the air gap of the device.^4–6^

In general, an effective way to increase the thermal conductivity of the TIM matrix is to fill the TIMs with fillers having high thermal conductivity. Among the many thermally conductive fillers, boron nitride (BN) is a ceramic material used for many TIMs and heat-radiating fillers. BN has a high thermal conductivity, making it a suitable filler for TIMs.^7,8^ Furthermore, it has a low coefficient of thermal expansion and high electrical insulation over a wide temperature range. In addition, BN is chemically stable in contact with most metals, waste, organic solvents, and polymers. However, the chemical stability of BN is also a disadvantage when it is used as a TIM filler. This is due to the low interfacial adhesion between the polymer matrix and BN particles, which are the raw materials of TIMs, because the high chemical stability of BN means that bonds are not easily formed. These low interfacial adhesion forces can create voids between the BN particles and the matrix inside the TIM complex, resulting in high thermal resistance and reduced mechanical strength. Therefore, by improving the interfacial affinity between the filler and the matrix through the surface treatment of the BN particles, higher performance TIM composites can be produced.^9–11^

We used the aniline trimer (AT) for surface treatment and dispersion. For the surface treatment, AT was dispersed with h-BN in an organic solvent, and the AT adhered to the dispersion of agglomerated BN, attaching itself to the surface of the plate-like BN particles. We posit that the dispersion is stabilized by strong π–π interactions between the aromatic rings in AT and h-BN. The AT surface treatment reduces the surface free energy between BN and the polymer matrix, resulting in increased conformity of BN with the polymeric material and enhanced thermal conductivity of the final composite.^12,13^

We report the utilization of AT as a noncovalent dispersant for h-BN. Sufficiently strong π–π interactions caused physisorption between the aromatic ring in AT and h-BN, which were then blended in an epoxy-terminated dimethylsiloxane (ETDS) matrix. We varied the AT weight ratio to determine the optimum amount of AT required to obtain high-performance composites in terms of the thermal conductivity, tensile strength, and storage modulus. By adjusting the amount of AT used in the surface treatment, we determined the optimal amount of AT for the surface treatment of BN.

In this work, the presence of AT was confirmed in the AT-treated h-BN and the samples were characterized using Fourier transform infrared spectroscopy (FT-IR), thermogravimetric analysis (TGA), and field emission scanning electron microscopy (FE-SEM) to determine the bonding, pyrolysis behavior, and morphology, respectively. The influence of surface treatments on the thermal conductivity of ETDS/AT-BN composites was investigated by laser flash analysis (LFA). In addition, we measured the storage modulus and tensile strength of the composites using dynamic mechanical analysis (DMA) and a universal testing machine (UTM).

## 2. Experimental

### 2.1 Materials

Hexagonal BN (ESK Ceramics/3M, Germany, ≥99%) powder with the plate size of 12–15 μm was used in this study. Aniline (C_6_H_5_NH_2_, ≥99%), *p*-phenylenediamine (C_6_H_8_N_2_, ≥99.9%), and ammonium persulfate ((NH_4_)_2_S_2_O_8_, 98.0%) were supplied by Daejung Science (Republic of Korea) and used to prepare the aniline trimer. ETDS was obtained from Shin-Etsu Silicon (KF-105, equivalent weight = 166.6 g eq.^−1^, density = 1.20 g cm^−3^; Republic of Korea) and was used after being thoroughly dried under vacuum at 60 °C for 24 h. 4,4′-Diaminodiphenylenemethane (DDM ≥ 99%), prepared by TCL Korea, was used as the curing agent without further purification. Sodium hydroxide (NaOH, ≥99%), ethanol (C_2_H_5_OH 70%), hydrochloric acid (HCl, 20%), and ammonium hydroxide (NH_4_OH, 30%) were obtained from Daejung Chemicals (Republic of Korea).

### 2.2 Synthesis of ETDS

Synthesis was carried out following previously reported methods.^14–16^ The mass ratio of the curing agent used to cure the epoxy was optimized to achieve a flexible ETDS matrix. In this study, the equivalent weight ratio of ETDS to DDS was fixed at 5 : 1. DDM (2 g) was added to a three-neck round bottom flask equipped with a reflux condenser pre-heated to 90 °C. After the addition of the ETDS resin (10 g), the mixture was heated in an oil bath at 90 °C under an N_2_ atmosphere for 1 h. The resulting mixture was kept in a vacuum oven at room temperature for 1 h to remove air bubbles inside the mixture. The mixture was then placed in an oil bath at 60 °C for 30 min under an N_2_ atmosphere. Final degassing was performed in a vacuum oven at room temperature for 1 h to remove residual bubbles.

### 2.3 Synthesis of aniline trimer

The aniline trimer was prepared following previously reported methods.^12,13,17^ Aniline and *p*-phenylenediamine were dissolved in a 1.0 M HCl solvent in an ice bath at 0 °C. A solution of ammonium persulfate in 1.0 M HCl was added to the solution for 30 min, and the reaction solution was stirred in an ice bath for 1 h. After the reaction, the product was filtered, washed with an HCl solution, and then cooled to 0 °C to collect the crude aniline trimer. The solid product was washed several times with 10% NH_4_OH solution and deionized (DI) water and then washed with HCl. Finally, it was dried in a vacuum oven at 60 °C for 24 h. The aniline trimer was obtained as a dark blue solid.

### 2.4 Surface treatment of BN using AT

After the completion of the organic synthesis and drying, AT was used for the surface treatment of BN particles at different mass ratios. AT treated BN particles were prepared at BN to AT ratios of 1 : 1, 2 : 1, 3 : 1, 5 : 1, 10 : 1, and 20 : 1, denoted AT-BN_1, AT-BN_2, AT-BN_3, AT-BN_4, AT-BN_5, and AT-BN_6, respectively. To achieve an even dispersion of BN and AT, the dispersion was evenly distributed *via* sonication in an ethanol solution for approximately 2 h before the surface treatment. The dispersed AT and BN were then treated by stirring for 2 h in distilled water and dried using a vacuum aspirator and a vacuum oven at 60 °C for 24 h.

### 2.5 Characterization

The FT-IR spectra were measured between 4000 and 400 cm^−1^ at 4.0 cm^−1^ resolution using an FT-IR spectrophotometer (PerkinElmer Spectrum100, USA) at room temperature. The FT-IR spectra were used to investigate changes in the structure of hexagonal BN. FE-SEM (SIGMA, Carl Zeiss) was used to investigate the morphologies of the BN particles and the composite cross-sections. The pure and surface-modified BN particles were analyzed by X-ray photoelectron spectroscopy (XPS, VG-Microtech, ESCA2000) with a Mg K_α_ X-ray source (1253.6 eV) and a hemispherical analyzer. During the curve fitting, the Gaussian peak width remained constant in each spectrum. The thermal conductivity of the composite at room temperature is given by *K* = *α* × *ρ* × *C*_p_,^18,19^ where *K*, *α*, *ρ*, and *C*_p_ are the thermal conductivity (W m^−1^ K^−1^), thermal diffusivity (m^2^ s^−1^), density (kg m^−3^), and specific heat capacity, respectively. An LFA (Netzsch 467 NanoFlash) instrument was used for the thermal diffusivity analysis. After measuring the thermal diffusivity, the specific heat capacity (*C*_p_) was measured at room temperature using a differential scanning calorimeter (DSC, Netzsch DSC 200F3). The storage moduli of the ETDS and the composites were determined by DMA (SS6100, Seiko Instruments, Japan) at a constant frequency of 1 Hz. Before analysis, samples were dried in a vacuum oven at 150 °C for 1 h to remove moisture.

## 3. Results and discussion

**Scheme 1:**
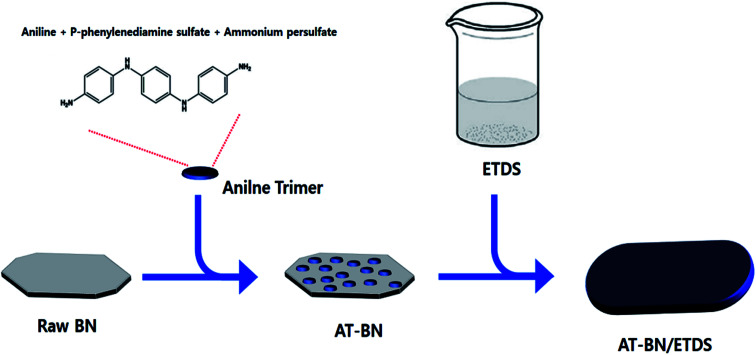
A schematic diagram of AT manufacturing and BN surface treatment.

**Fig. 1:**
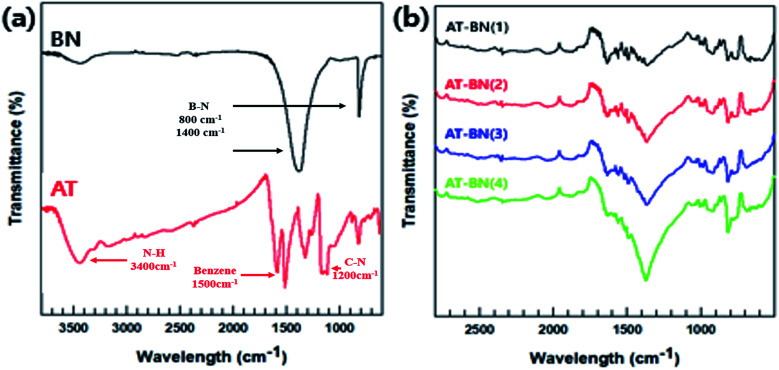
FT-IR spectra of raw BN, AT (a), and AT-BN_(1–4) (b).

As schematically illustrated in Scheme 1, AT is adsorbed on the h-BN surface. One possible reason for this physisorption is the π–π interactions between the h-BN particles and AT. Hexagonal BN has a sheet structure held together by attractive van der Waals interactions, and aromatic AT can form π–π interactions with the aromatic sheets of hexagonal BN. We performed FTIR spectroscopy studies to confirm the chemical structure of the as-synthesized AT, BN, and AT-BN. Fig. 1 shows the FT-IR spectra of pristine BN, pristine AT, and AT-BN. The FTIR spectrum of BN contains strong vibrational bands between 800 and 1400 cm^−1^. In the case of pure AT, there are multiple sharp peaks at 1596, 1504, 1300, and 830 cm^−1^, which confirm the successful synthesis of the aniline trimer.^20^ As shown in the FT-IR spectra of the AT-BN_*n* samples (*n* = 1–6), the vibrational bands of AT and BN overlap, which makes analysis difficult. Nevertheless, a vibrational band corresponding to the phenazine structure has been observed previously,^21^ indicating the existence of π–π bonding interactions between the h-BN surface and AT. Furthermore, as the amount of AT increased, there was a remarkable increase in the intensities of peaks corresponding to AT. In particular, when the BN : AT ratio exceeded 3 : 1 (25%), a peak corresponding to AT that had not been observed previously because of the overlap of the high-intensity BN peaks was observed in the FT-IR spectrum.

**Fig. 2:**
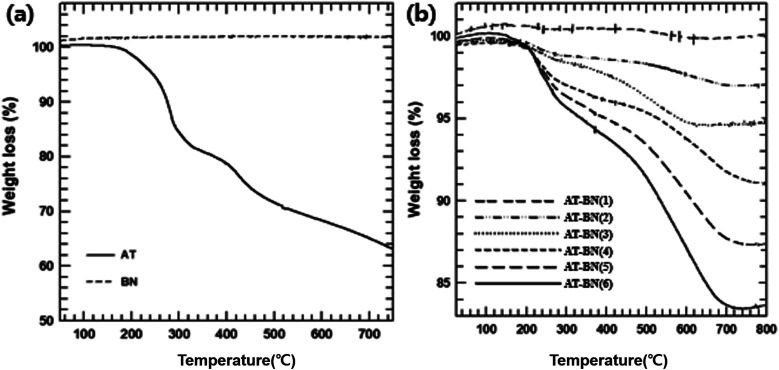
TGA curved of raw BN, AT (a), and AT-BN_(1–6) (b).

Because the thermal stability of AT-BN could be affected during extrusion at high processing temperatures, the weight changes of pure BN, pure AT, and AT-BN_1, AT-BN_2, AT-BN_3, AT-BN_4, AT-BN_5, and AT-BN_6 were quantified by TGA analysis. Fig. 2 shows the thermograms of pristine BN, as-synthesized AT, AT-BN_1, AT-BN_2, AT-BN_3, AT-BN_4, AT-BN_5, and AT-BN_6. The TGA measurements were performed at a heating rate of 10 °C min^−1^ up to a maximum temperature of 800 °C under a nitrogen atmosphere. Under these conditions, weight loss was not observed at temperatures up to 800 °C for pristine BN. Because BN is a ceramic filler with excellent heat resistance, thermal decomposition does not occur at high temperatures. However, AT and AT@BN (1 : 1–20 : 1) showed weight losses arising from thermal decomposition, and we observed multiple thermal degradation processes. The first stage is the decomposition of the amine group (between 250 and 300 °C), and the second stage is the decomposition of the benzene ring (approximately 400 to 450 °C). Furthermore, we confirmed that the degree of thermal decomposition increased with increasing amount of AT. For example, the difference in the pyrolysis of AT-BN_6 sample (with only 4 wt% AT) compared to that of pristine BN was negligible. On the other hand, the highest weight loss was observed for AT-BN_1, in which the AT weight content is half the total mass. This indicates quantitative removal of AT from the BN surface. Qualitative analysis of the surface-treated AT is possible using TGA analysis. As shown in Fig. 2(a), when pristine AT was pyrolyzed at 800 °C, it lost 62% of its mass. In addition, AT-BN_1, AT-BN_2, AT-BN_3, AT-BN_4, AT-BN_5, and AT-BN_6 showed reduced weight ratios of 83.9%, 87.5%, 91.1%, 95.5%, 97.0%, and 99.9%, respectively. From our calculations, we confirmed that each sample lost the same amount of weight as the amount of AT added to prepare the AT-treated BN.

**Fig. 3:**
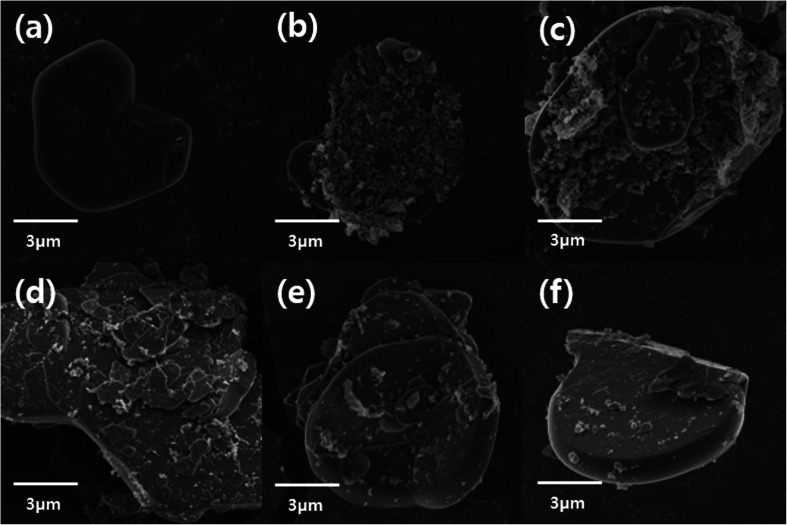
FE-SEM image of raw BN particles (a), AT-BN_1 (b), AT-BN_2 (c), AT-BN_3 (d), AT-BN_4 (e), and AT-BN_5 (f).

The surfaces of the hexagonal BN particles were observed by FE-SEM for pristine BN, AT particles, and AT-treated BN. The mean size of the BN particles is approximately 12–15 μm. The presence of platelet-shaped AT particles on the surface of BN is evident in Fig. 3. The AT-treated BN shows the presence of nano sized AT on the surface. Combining the FTIR and TGA data and the FE-SEM images, the amount of AT attached to the surface of the BN particles increases with increasing weight ratio of AT. All the AT-treated BN samples show the presence of higher amounts of AT on the BN surface with increasing AT weight ratio. However, the BN particle surfaces of AT-BN_1 and AT-BN_2 does not show much difference. As the amount of AT increases, not all the AT is attached to the surface of the BN particles by π–π bonding. The AT not attached to the surface of BN become aggregated through π–π bonding between the ATs. This phenomenon occurs even at low amounts of AT, but it is prominent in samples containing 40 wt% AT. Fig. 3 shows the FE-SEM images of the aggregated AT particles in samples AT-BN_1, AT-BN_2, AT-BN_3, and AT-BN_4. It is evident that aggregation occurs even at low AT ratios; however, the quantity of aggregated AT particles and their sizes are very small. In particular, the AT-BN_1 sample shows the presence of large amounts of aggregated particles, indicating that the amount of AT in this sample exceeds that required for the surface treatment of BN.

**Fig. 4:**
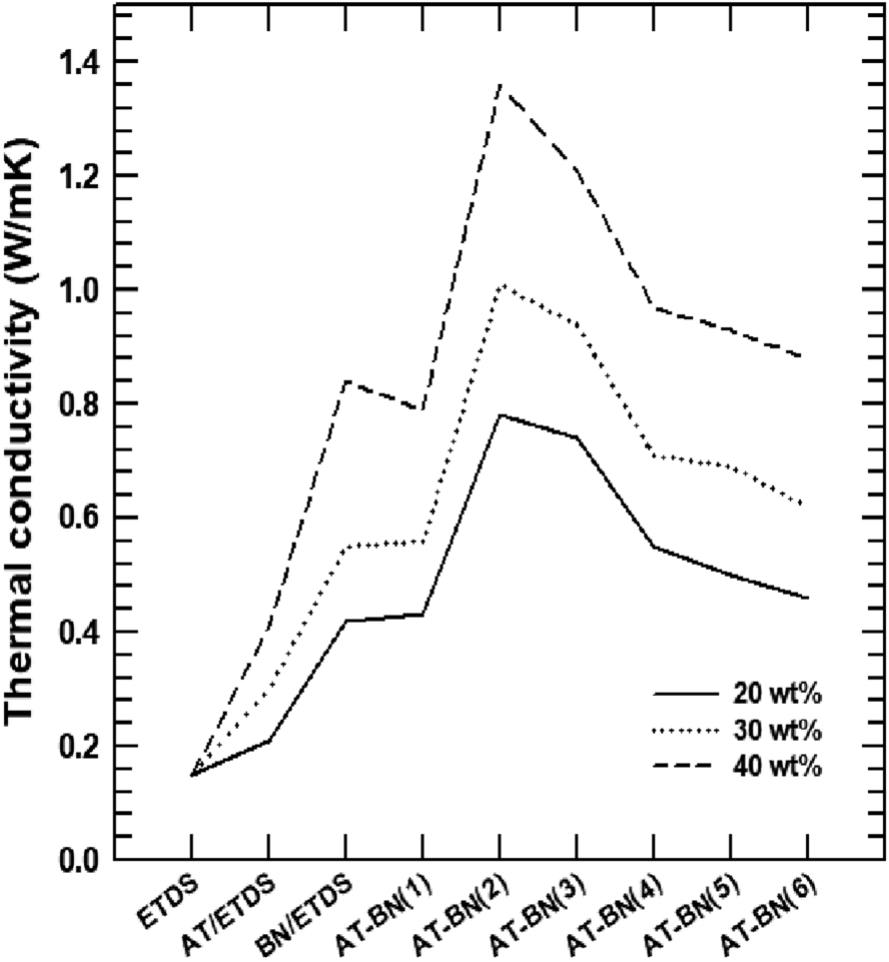
Thermal conductivity from LFA data of raw ETDS, BN@ETDS, APBN@ETDS, ATBN@ETDS, and APATBN@ETDS.

**Fig. 5:**
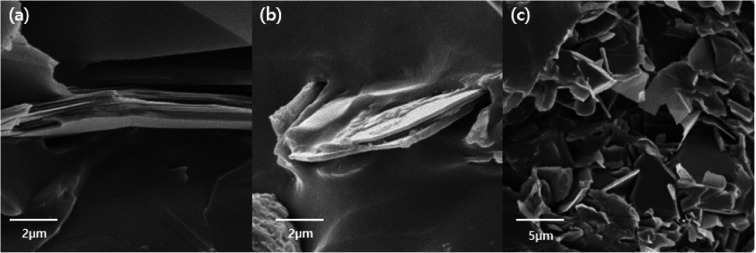
Cross-sectional image raw BN/ETDS (a), AT-BN_2/ETDS (b), and AT-BN_1/ETDS (c).

Fig. 4 shows the thermal conductivity plots of the composites containing pure ETDS, pristine BN/ETDS, pristine AT/ETDS, AT-BN_1, AT-BN_2, AT-BN_3, AT-BN_4, AT-BN_5, and AT-BN_6 at 50 wt% filler contents. Pure ETDS exhibits a low thermal conductivity value of 0.19 W m^−1^ K^−1^, which is in agreement with previously reported values. The increase in thermal conductivity was observed after adding a conductive filler to the sample, indicating an increase in the thermally conductive tunnel for phonon scattering. The thermal conductivity of pristine ETDS, BN/ETDS, pristine AT/ETDS, AT-BN_1, AT-BN_2, AT-BN_3, AT-BN_4, AT-BN_5, and AT-BN_6 composites increased to 0.15, 0.412, 0.843, 0.792, 1.358, 1.214 0.974, 0.930, and 0.881 W m^−1^ K^−1^, respectively, at 40 wt%. All the surface-treated samples exhibited higher thermal conductivity than the pristine BN/ETDS composites.^22,23^ We assume that the increased surface wettability between the ETDS matrix and the BN filler improves the thermal conductivity of the AT surface-treated BN composite, which reduces voids and provides bridges for heat conduction.^24,25^ However, the thermal conductivities of the composites did not increase at higher AT weight ratios. The BN/ETDS composite with the highest thermal conductivity is AT-BN_2, followed by AT-BN_3 and AT-BN_1. This is because the amount of AT added for the coating of BN becomes too large, and the excess AT is dispersed in the ETDS complex rather than on the surface of BN. The thermal conductivity of pristine AT is only 7.8 W m^−1^ K^−1^, which is higher than that of ETDS but significantly lower than that of BN. In addition, owing to the presence of the aggregated AT particles, the shape of the inside of the composite becomes irregular and interferes with smooth heat transfer. As shown in Fig. 5, the BN particles were well dispersed within the ETDS matrix. The AT coated on the BN surface improves the adhesion between the ETDS and BN, which results in smooth heat flow. However, the excess AT does not adhere to the BN surface and is distributed in the ETDS matrix. Significant aggregation and dispersion of AT were observed in the AT-BN_1 composite. Notably, the presence of AT coating on the BN surface and its dispersion causes serious drawbacks for TIM applications.

**Fig. 6:**
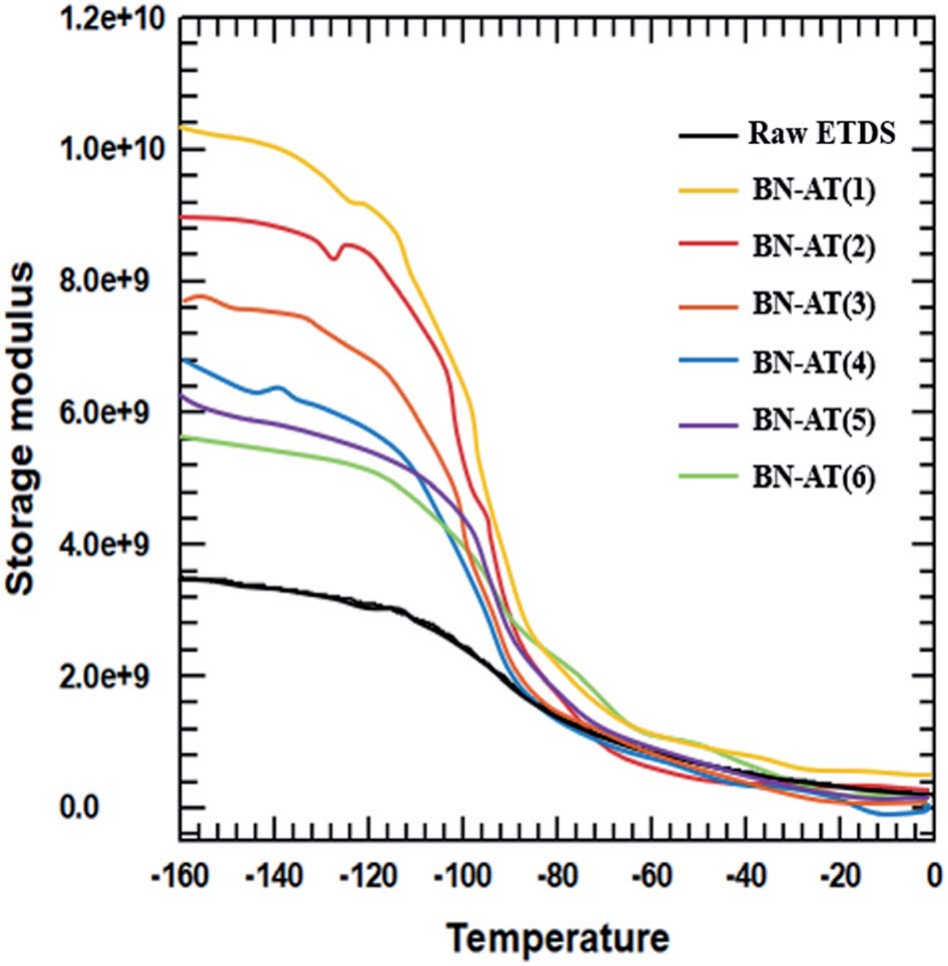
DMA analysis of the raw ETDS and the AT-BN_(1–6) composites.

**Fig. 7:**
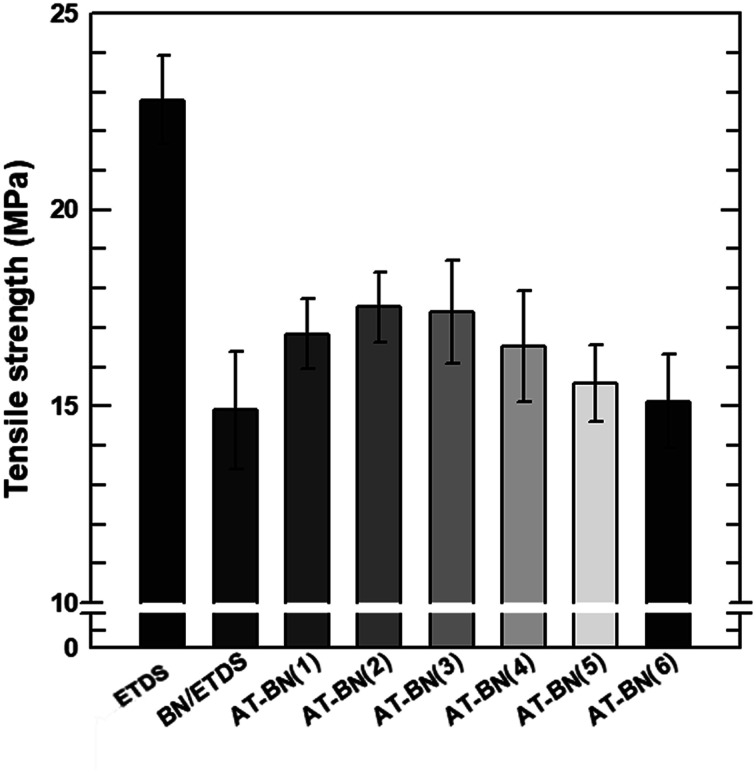
Tensile strength by UTM test of raw ETDS, BN/ETDS, and AT-BN_(1–6).

The storage modulus and tensile strength of the ETDS/BN composites were tested using a DMA and UTM, respectively. The storage moduli of the ETDS/BN composites are shown as a function of temperature in Fig. 6. The temperature range for the measurements was 30–150 °C. At all studied temperatures, the storage modulus of the ETDS composites increased in the following order AT-BN_1, AT-BN_2, AT-BN_3, AT-BN_4, AT-BN_5, AT-BN_6, pristine BN/ETDS. The AT-treated BN samples exhibited the greatest improvement, owing to the large of surface-modified area and high storage modulus. The increase in the storage modulus of the BN/ETDS complexes had a significant effect on the interfacial affinity between BN particles and ETDS matrix because the ETDS attached to the surface of the BN particles lose fluidity and this ETDS reduces the loss of storage elastic modulus within the composite.^18^ In addition, the AT not attached to the BN surface increases the storage modulus of the ETDS matrix. Therefore, the highest storage modulus was achieved for the AT-BN_1 composite. The flow loss after curing and curing increases the storage modulus. However, the tensile strength shows a different trend from that of the storage modulus. As shown in Fig. 7, as in previous studies,^22^ the tensile strength of a polymer decreases when a ceramic filler is added. The tensile strength was highest in the AT-BN_3 composite, and the AT-BN_1 composite exhibited a much lower tensile strength than the pristine BN composite. The tensile strength decreased with increasing filler content. However, the increase in the amount of aggregated AT with increasing AT content resulted in an increase in the tensile strength of the ETDS/BN composites by acting as a filler and not as a surface treatment agent.

## 4. Conclusion

In this study, the effects of surface treatment of BN with AT on the thermal and the mechanical properties of ETDS/BN composites were investigated. The chemical functionalities of AT-treated BN surfaces and the optimum amount of AT required for the best performance were revealed by FTIR, TGA, and FE-SEM studies. The AT-BN_2 composites exhibited the highest thermal conductivity. The highest value of thermal conductivity was 1.25 W m^−1^ K^−1^, which is 8.06-fold higher than that of pristine ETDS. The storage modulus and the tensile strength of the composites were tested using DMA and UTM, respectively. The storage moduli of the composites with surface-treated BN were higher than those of ETDS with pristine BN, and the tensile strength also increased upon AT treatment of BN. Finally, as the amount of AT increased, the thermal conductivity and the mechanical properties increased, and the amount of AT ratio to be used for the surface treatment of BN was analyzed to be excellent ratio is 2 : 1.

## Conflicts of interest

There are no conflicts to declare.

## Supplementary Material
